# Learning in and across communities of practice: health professions education students’ learning from boundary crossing

**DOI:** 10.1007/s10459-022-10135-5

**Published:** 2022-07-12

**Authors:** Malou Stoffels, Stephanie M. E. van der Burgt, Larike H. Bronkhorst, Hester E. M. Daelmans, Saskia M. Peerdeman, Rashmi A. Kusurkar

**Affiliations:** 1 Research in Education, Amsterdam UMC Location Vrije Universiteit Amsterdam, De Boelelaan 118, 1081 HZ, 1007 MB Amsterdam, The Netherlands; 2 Amsterdam UMC, VUmc Amstel Academy, Institute for Education and Training, Amsterdam, The Netherlands; 3 Teaching & Learning Centre (TLC) FdG, University of Amsterdam, Amsterdam Umc Location Amc, Amsterdam, The Netherlands; 4 Department of Education, Faculty of Social and Behavioural Sciences, Utrecht University, Utrecht, The Netherlands; 5 Faculty of Medicine, Department of Skills Training, Amsterdam UMC, Vrije Universiteit Amsterdam, Amsterdam, The Netherlands; 6 Department of Neurosurgery, Amsterdam UMC, Location VUmc, Amsterdam, The Netherlands; 7 LEARN! Research Institute for Learning and Education, Faculty of Psychology and Education, Vrije Universiteit Amsterdam, Amsterdam, the Netherlands

**Keywords:** Clinical education, Nursing education, HPE, Landscapes of practice

## Abstract

Learning to adapt to new contexts is crucial in health professions education (HPE). Boundaries between and within contexts challenge continuity in students’ learning processes. Little is known about how HPE students can make these “boundary experiences” productive for learning. We investigated how and what nursing students learn from boundary experiences while they are simultaneously growing into a community of practice (CoP). Using a boundary-crossing lens, experiences of discontinuity were identified in pre-placement and post-placement interviews and diary fragments with 14 nursing students during their placement in an academic hospital. We found that students experience discontinuity as a result of different approaches to nursing care and to learning, both between (academic and clinical) settings and within a setting. When students feel safe enough, they can convert boundary experiences into meaningful learning situations, such as critical discussions with staff. Successfully overcoming boundary experiences improves students’ understanding of healthcare and professional development and helps them to develop a personal approach to learning. Students critically address boundary experiences when they are motivated to learn and when they perceive a violation of ethical standards but not when they are concerned that it will affect their assessment. Objects designed to bridge theory and practice can generate additional barriers. This study adds to the HPE literature by demonstrating the learning potential of boundaries and to the broader literature by showing how responses to boundary experiences are intertwined with the process of growing into a CoP. The findings can be used to design future boundary objects.

## Introduction

Health profession education (HPE) purposefully includes multiple clinical placements for students to develop competence in different authentic communities of practice. While students learn the most from these placements, they typically experience uncertainty when starting a new clinical placement (van Dijk et al., 2017). They have to find their way into a new community of practice while feeling inadequately prepared by their theoretical training and experiencing a lack of connection with previous placements (Alzayyat & Al-Gamal, 2014; Gassas, 2021). Moreover, at any given time, the expectations of their current practice setting may conflict with school expectations or expectations from their earlier placements.

As such, learning to deal with uncertainty and adapting to new contexts is essential for HPE (Mylopoulos et al., 2018; Weeks et al., 2017) and subsequent professional practice. The strategies that students adopt to learn from these boundaries may be predictive of their future success. However, there is a gap in the literature regarding whether and how HPE students learn from boundary experiences, how they experience support by their educational program, and how this learning is affected by their position within the community of practice (Hodson, 2020; Stoffels et al., 2019). Therefore, in this study, we explored how HPE students respond to these boundary experiences and how this affects their learning. Insight into these mechanisms may help prepare and support students throughout their learning trajectories.

Several studies have provided insight into how HPE students’ novel position within a community of practice affects their learning. Clinical placements offer students the opportunity for competence development and to socialize into the profession (Salisu et al., 2019). During placements, students strive to move from a peripheral to a fully participating position in the community of practice (Cruess et al., 2018). Team members can facilitate this process and benefit from it through mutual learning (Thrysoe et al., 2010). Growing into a community is a prerequisite for competence development. When students lack a sense of belongingness to the team in which they are posted, especially in shorter placements, they focus on fitting in instead of learning (Bernabeo et al., 2011). Misalignment between the goals of the students and the community of practice can give rise to tensions and uncertainties (Olmos-Vega et al., 2019). Eventually, this may hamper the development of higher order competence, which is necessary for becoming future advocates of the profession, such as critically questioning clinical practice (Levett‐Jones and Lathlean, 2009; Bernabeo et al., 2011; Henderson et al., 2012).

In addition to the process of moving toward the center of each community of practice, students’ competency development involves integrating what they learn across contexts (i.e., schools and different placement settings). This process is far from linear. Different contexts offer fragmented experiences (Hirsh et al., 2007; Liljedahl et al., 2015; Roxburgh et al., 2012) and students experience a gap between theory and practice (Greenway et al., 2018; Peters et al., 2017). Students struggle to make connections between different contexts of training spontaneously (O'Brien et al., 2007). Educational institutions and hospitals, therefore, collaborate to smoothen students’ transitions between contexts through the exchange of staff and expertise between settings, tools such as portfolios, and simulation-based education (Berndtsson et al., 2020; Brown et al., 2019; Peters et al., 2017). Moreover, educational models are designed in which traditional block rotation models are replaced with models in which students follow a cohort of patients across disciplines (Worley et al., 2016).

While much research has focused on the integration and alignment of learning experiences, as well as fitting into each single community of practice, recently, the learning potential of boundary experiences across contexts has gained attention. The boundary-crossing perspective is inspired by theories of situated learning and expansive learning. It has been shown that ongoing action or interaction (such as learning or working) can be temporarily hampered by sociocultural differences between contexts or between agents within a context, but re-establishing ongoing action (i.e., continuity) can be a resource for learning (Akkerman, 2011; Akkerman & Bakker, 2011, 2012; Bronkhorst & Akkerman, 2016; Jacobs, 2017). In healthcare education, a *landscapes-of-practice* model has been suggested as an alternative to the traditional community of practice (CoP) model. This model acknowledges how development does not entail a single journey toward the center of a (single) community of practice but a trajectory over multiple communities. Learning across communities of practice stimulates the development of complex learner identities and knowledgeability but also brings new knowledge into each community (Hodson, 2020; Wenger-Trayner & Wenger-Trayner, 2014). Discomforting or unexpected experiences encourage students to question practice, develop their professional identity, and prepare students for independent practice in an ever-changing profession (Dornan et al., 2014; Mylopoulos et al., 2018; Teunissen & Westerman, 2011). However, little empirical work exists about how learning at the boundaries takes place within health profession education (Hodson, 2020).

Boundary crossing literature differentiates between vertical learning processes (the trajectory toward becoming an expert within a field) and horizontal learning processes, which occur across parallel contexts that may apply different criteria of knowledge and skill (Engeström et al., 1995). In a review of the boundary crossing literature, Akkerman and Bakker (2011) have described four horizontal learning mechanisms: identification, coordination, reflection, and transformation (see Table 1 for a description of learning mechanisms and an example from the HPE context). These learning mechanisms re-establish continuity in action and interaction across boundaries without removing the boundary. That is, removing differences between contexts is considered impossible, but also undesirable, given the unique, historically developed characteristics of each specific context and hence their value for learning and working. Learning mechanisms can take place at the organizational level, as well as at the interpersonal and intrapersonal levels. They can take place between contexts, between groups and between individuals and/or positions within a context (Akkerman & Bruining, 2016). Learning mechanisms can be supported by boundary objects and brokers. Boundary objects are tools, such as portfolios, designed to fulfill a bridging function across contexts. Brokers, such as mentors or practice educators, engage in (translating) activities to connect practices (Barry et al., 2020). Although horizontal learning mechanisms at the boundary do not represent consecutive stages, previous studies have reported a development in learning mechanisms, with initial efforts toward coordination, followed by reflection and transformation mechanisms (Akkerman & Bruining, 2016). Previous work in healthcare education has suggested that a focus on identification and coordination could keep learners from transforming their professional identity (Hazen et al., 2018). Thus, these learning mechanisms provide a useful language to study and describe how learning at the boundaries takes place (Akkerman & Bruining, 2016; Hazen et al., 2018; Mesker et al., 2018). Although learning from boundary experiences and growing into the community of practice occur simultaneously, little research has been done on how these processes co-occur and intertwine.

**Table 1:** Learning mechanisms adapted from (Akkerman & Bakker, 2011) with examples in Health professions education

Learning mechanisms	Description (at the intrapersonal level)	Example from HPE
Identification	Becoming aware of and defining different practices in relation to each other, leading to renewed insight into what the diverse practices concern	e.g., identifying how theory differs from practice, how health practices differ
Coordination	Searching for effective means and procedures that allow working across diverse practices efficiently	e.g., discussing how competence can be operationalized in a practice setting
Reflection	Coming to realize and explain differences between practices and thus to learn something new about their own and others’ practices	e.g., experiencing how protocols learned at school are too time consuming for complex health care
Transformation	Shaping of new practice or identity	e.g., contributing to a change of clinical practice in alignment with school theory or developing a professional identity

Nursing education makes an interesting case for studying how HPE students make sense of boundary experiences across settings. In nursing education, students alternate between school and practice settings from the first year of their training onward. In practice periods of several months, they work toward a high level of independence. Within these placements, they work alongside different supervisors with varying approaches to nursing care and education (Liljedahl et al., 2015).

**Fig. 1:**
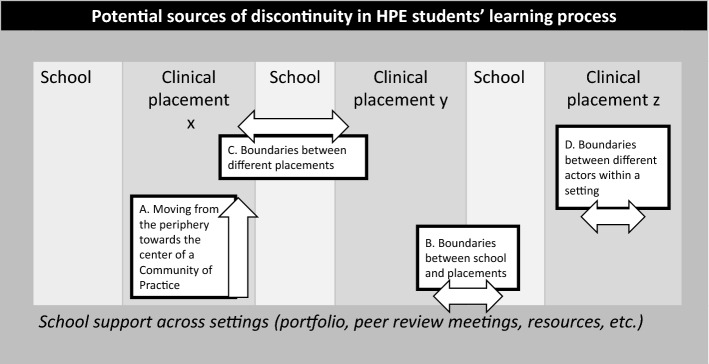
Potential sources of discontinuity in HPE students’ learning processes

Thus, in each clinical placement, students’ learning processes can be temporarily hampered (i.e., discontinuity) by having to: a) move from the periphery toward the center in a novel community of practice, b) enact knowledge and skills as learned in school, c) adapt experience from previous placements in a novel, different context, and d) deal with differences in practice within the same context. See Fig. 1 for potential sources of discontinuity HPE students cross throughout their training that are described in the literature.

This study aims to understand how students’ responses to boundary experiences, their learning and professional development and their process of growing into a community of practice are interwoven during different stages of their clinical placements. The findings of this study may help us understand how learning takes place in the landscapes of practice and how nursing students can be supported to optimally benefit from the boundaries they encounter during and after training.

The main research question is as follows:

- How does nursing students’ behavior in response to boundary experiences impact their learning and professional development at different stages of a clinical placement, while they simultaneously grow into the community of practice?

To answer this question two sub questions will be addressed:

- What boundary experiences and peripheral experiences do nursing students report during their clinical placements?
- What learning mechanisms occur in nursing students’ responses to different boundary experiences?

## Methods

### Study design

This study employed a longitudinal qualitative approach. Data were collected in three phases from nursing students: (a) pre-placement interviews before they started their clinical placement (shortly before or after their introduction to the ward), (b) solicited (audio) diary entries during the placement, and (c) post-placement interviews in the last week of the clinical placement.

Pre-placement interviews and diary entries were used to (a) increase students’ awareness of potential boundary experiences from right before the start of the clinical placements, (b) give students the opportunity to capture and reflect on these experiences immediately following their occurrence and (c) allow the researcher to ask directed questions in the post-placement interviews.

### Reflexivity

We work within a constructivist paradigm in which knowledge is constructed through interactions between researchers and participants (Bergman et al., 2012). Diary fragments allowed to capture real-time experiences in which the researcher’s voice was minimized (Filep et al., 2018). Post-placement interviews allowed for validation of the fragments. Diversity in the backgrounds and occupations of the research team limited personal or disciplinary bias (Giacomini & Cook, 2000): MS is trained in psychology and education and works as an educational consultant and researcher in nursing education, SMEB is trained in sociology and works as a researcher in postgraduate medical education, LHB is trained in educational sciences and works as an assistant professor in education with expertise in boundary crossing, HED is trained in medicine and works as a head of the master’s program in medicine, SMP works as a neurosurgeon, professor in education and vice dean, RAK is trained as a medical doctor and works as an associate professor in medical education. All authors are trained and have experience in qualitative research. Trustworthiness was established by transparency in the research process as well as by regular team discussions. The Ethical Review Board of the Netherlands Association for Medical Education granted ethical approval (NVMO, file 2019.5.3). All participants provided informed consent. The Standards for Reporting Qualitative Research (SRQR) guided our reporting (O'Brien et al., 2014).

### Setting

We conducted this study in an academic medical center in the Netherlands. The hospital offers undergraduate students clinical nursing placements (10–40 weeks), for which theoretical training is provided by higher education partners. Students work toward competency achievement based on individual learning plans and goals. Practice curricula are designed in collaboration between educational advisors and teachers/supervisors working in the practice. In clinical practice, students work alongside several clinical supervisors who are registered nurses with basic training in supervisory skills. Additionally, each nursing ward has a clinical educator who is exempted from patient care to coordinate clinical training and coach students and clinical supervisors. Some nursing wards are modelled as “dedicated educational units” (Williamson et al., 2020). The hospital has a quality management system in which the educational climate is constantly monitored and improved.

### Participants and sampling

Undergraduate nursing students with previous nursing experience in wards or settings other than the current clinical placement (previous clinical placements and/or work experience) were eligible for participation in the study.

The secretary of the clinical nursing education department sent an information and recruitment email to all eligible students who were going to start a clinical placement at Amsterdam UMC. The main researcher introduced the study on an introductory day at the hospital. Recruitment was repeated in the following semesters until we reached data sufficiency. All students who met the inclusion criteria and were willing to participate were invited by the main researcher. One participant dropped out of the study before finishing the clinical placement and was excluded from the analysis. Two participants failed to provide diary entries, one because of reported technical problems and one as a result of stress caused by the beginning of the covid-pandemic. Because both agreed to participate in the final interview, they were included in the analysis. See Table 2 for demographics and number of diary entries.

**Table 2:** Demographics of participants and number of diary entries

Pseudonym	Round of data collection	Age	Study year	Number of diary entries
Anne	1	23	3	4
Jenny	1	22	2	2
Kimberley	1	23	3	1
Jack	1	26	3	6
Abigail	1	21	2	–*
Denise	2	22	3	3
Alexis	2	22	4	2
Kayla	2	19	2	3
Luna	2	18	2	15
Silvia	2	21	4	21
Gemma	2	20	3	4
Ahmed	3	22	1***	2
Rosalyn	3	22	3	–**
Phoebe	3	23	3	1

^*^Participant commented that she could not send diary entries due to technical difficulties

^**^Participant commented she was not able to send diary entries as a result of large stress caused by the beginning of the covid−pandemic

^***One first^^−^year student was allowed to participate because of prior work experience in nursing

### Data collection

Data collection took place between February 2019 and July 2020. MS conducted a pilot interview for the pre-placement interview with a clinical nursing educator to adjust the interview protocol. The interviews took place in a private room in the hospital outside the ward and lasted for around 60 min. Due to Covid-19 restrictions, two of the 14 post- placements interviews were held online via MS Teams. The interviews were audiotaped and transcribed verbatim by a research assistant.

*Pre-placement interviews:* MS conducted individual semi-structured interviews about students’ background, experience with nursing education and clinical placements, expectations of the clinical placement, self-concept as a nurse and image of what a nurse should be like. After the start of the interview (averaging 30 min), MS introduced the procedure for (audio) diaries to the students. Before each interview, the participants were informed that participation was voluntary, and that participation would have no bearing on grades or progression.

*(Audio)-diaries:* During the pre-placement interview, MS gave students instructions to report a) events in which they encountered something that was different from what they had learned or experienced before, b) their subjective experience of this event, and c) their subjective behavior. To point students’ attention towards boundary experiences, a prompt sheet for the diaries was offered (Verma, 2021). Apart from that, students were free to choose their own form (length of text, to whom the diary entries were directed etc.). To minimize the burden on students, they were allowed to keep diary entries short and to only write or record something when they felt that had a boundary experience. They did not receive feedback or follow up questions on diary entries before the post-placement interview. In the first round of data collection, participants were asked to make audio recordings. As a result of participants’ comments and after additional ethical approval, participants could choose between written and spoken diary entries. Participants sent their diary fragments to the main researcher via encrypted safe-send software (SURFfilesender). After receiving a fragment, the main researcher thanked the students and encouraged them to continue, but did not give any content feedback. Upon request, she repeated the instructions or confirmed that diary fragments met her expectations. MS followed up with the students around every four weeks if they failed to report. Most diary fragments included short answers to the questions on the prompt sheet; some students gave more elaborate accounts of their (boundary) experiences and reflections on those. Individual diary entries ranged from 34 to 256 words.

*Post-placement interviews:* MS conducted individual semi-structured interviews about students’ experiences in the clinical placement, facilitators/barriers, their own learning behavior, events in which they experienced something that was different from what they learned or experienced before and (self) concept of a nurse. Information from pre-placement interviews was used as a prompt to elaborate on boundary experiences in the current placement in light of previous experiences. Where necessary, clarification was asked for diary entries. When the diary entries were short and the reflections appeared somewhat superficial, they were discussed more in-depth during the interview.

## Analysis

Analysis involved several stages of inductive and deductive coding. To explore and categorize the different kinds of boundary experiences appearing in this context, we used open coding. In order to analyze students’ learning mechanisms in response to those boundary experiences, the learning mechanisms identified by Akkerman and Bakker (2011) were used. Using previously identified learning mechanisms as a framework, allowed us to bring depth to our interpretation and eventually compare our findings with previous literature. Finally, different type of results were integrated into three overarching themes to answer our main research question.

In the first phase of data analysis, MS and SMEB undertook in-depth reading of the transcribed pre-placement interviews, (audio) diary fragments, and post-placement interviews. Pre-placement interviews were mainly used to provide input for post-placement interviews. However, they were included in the analysis for discontinuities and responses to those, experienced in the preparation or first acquaintance (e.g., an introductory email) with the clinical placements. Variations in frequency and interval between (audio) diaries did not allow for true longitudinal analysis other than the participants’ own reports of development in experiences (Verma, 2021).

MS first coded all data for boundary experiences and peripheral experiences. Boundary experiences were operationalized as differences encountered across and within contexts that lead to “discontinuity” and discontinuity as the (temporary) disruption or hampering of ongoing learning processes. Peripheral experiences were operationalized as negative experiences of growing into the community of practice that lead to discontinuity. Additionally, MS grouped boundary experiences into qualitatively different subgroups using open coding. SMEB independently coded five of the transcripts for boundary experiences and peripheral experiences. MS discussed coding with SMEB to cross-check for trustworthiness and consistency and with LHB for accuracy within the boundary framework until consensus was reached.

Next, MS deductively used Akkerman and Bakker’s (2011)descriptions of the four learning mechanisms to code mechanisms through which boundary experiences impacted students’ learning and professional development. Coding was again discussed with SMEB for consistency and with LHB for accurate interpretation of the learning mechanisms.

After consensus was reached, MS went back to the transcripts to map the relationships between (a) boundary experiences (b) learning mechanisms (c) peripheral experiences (d) antecedents/consequences (e) references to time and phase of training. Based on the charted data MS identified three dominant themes that were discussed with all authors and refined iteratively with repeated consultation of the data.

MS compared the frequency of codes in the data of each participant to look for patterns in the co-occurrence of codes. However, no clear patterns can be distinguished.

## Results

### Types of boundary experiences and learning mechanisms.

All students reported boundary experiences related to (a) differences in how patient care is delivered/nursing care is understood, as well as to (b) differences in how students’ learning process is understood and supported Not only did they experience differences between settings, but also between supervisors within a single setting. Some of the boundary experiences could not be traced back to a single boundary but involved a difference between current practice and students’ professional values, based on their integration of previous experiences. All four learning mechanisms as identified by Akkerman and Bakker (2011) (identification, coordination, reflection, transformation) were found in response to both types of boundary experiences. On top of boundary experiences, all students reported some negative experiences related to their peripheral position within the community of practice. See Appendix 1 for types of boundaries, boundary experiences, learning mechanisms and peripheral experiences.

*How does nursing students’ response to boundary experiences impact their learning and professional development, while they simultaneously grow into the community of practice?*

We identified three themes that answered our main research question:

- Experiencing theory in action: Integrating resources and engaging in interprofessional conversations
- Contrasting role models: working toward professional identity development
- Structuring the learning process: translating requirements and recognizing individual learning preferences

### Experiencing theory in action: Integrating resources and engaging in interprofessional conversations

At the start of their clinical placement, the students were confronted with the fact that previously acquired knowledge and skills were insufficient and/or not readily applicable to the current setting. This hampered the students’ self-confidence and development of independent practice. However, they accepted it as a fact and often thought that you learn it in practice anyway. They initially tried to overcome this discontinuity by *coordinating resources*. They collected information on the ward, such as protocols and patient folders, and took their time on and off the ward to study patient records. They used school assignments and formats to integrate this new knowledge with what they had previously learned.

Identifying the unique body of knowledge underlying patient care on the ward helped them engage in meaningful interactions with staff, both disciplinary and interdisciplinary, resulting in knowledge they would not have acquired otherwise:

*“Well, I just asked the doctors a lot of questions because they could explain it very well, very logical. That you know certain things about anatomy and physiology, and then go into it much more deeply and link it to the current clinical picture. And you can't really find that on the internet or in books.” (Silvia).*

The exchange of knowledge and resources with other peers and supervisors leads to an increased understanding of complex patient cases for everyone involved.

The students realized that theory and practice were two different things. Experiencing how knowledge and skills worked out in practice initially helped them i*dentify the* inherent differences between school and practice. Subsequently, it made them *reflect on* how they could get more out of their school preparation:

*“Yes, with hindsight and looking back, the basics, the theory, are useful in some way. And yes, in school when you do it, just like with communication skills, now you understand the theory behind it much better, but when we had to do that at the time, it was a big joke actually for us. …but I do think we will all take it a little more seriously than we did back then” (Kayla).*

As long as students felt they were given room to learn at their own pace and to ask questions, they successfully overcame discontinuities in terms of knowledge and skills. However, they did suggest that some specific training around the ward could have facilitated this process. Some students attempted to *transform* existing practices. The duration of the placement usually imposed restricitons on the impact students felt they could have on the organization of learning for themselves or for future students:

*“I had argued for a short clinical lesson about what kinds of lab tests are taken here often, but in the end nothing was done about it. And it was almost at the end of the placement then, so that didn't help us much anymore. But I think for the next group, I also included that in the feedback for my clinical educator, on such a theme day, there should also be a brief presentation about what kinds of lab tests there are. That's useful”. (Silvia).*

Occasionally, students felt that actual nursing practice should more structurally be aligned with theory. Although the students’ critical voices were appreciated, their positions as students often hindered actual *transformations.*

*“I also found it fun to find out certain things about how things could possibly be better or why things actually go a certain way, that seemed fun too. That you don't settle for how it’s always done and so be it. So I wanted to do something with that later on in my placement, but unfortunately that was not possible. ….It's certainly not that I was inhibited in my acting, but it is of course-…It was just a bit of hey, pay attention to the basics and what you're already doing outside the basics it is very good, but it's not the main goal.” (Ahmed).*

### Contrasting ‘role models’: working toward professional identity development

The students faced the fact that nurses worked in a different way from how they had perceived it before or from how they had learned it at school. Moreover, they recognized different practices *within* a setting. These differences ranged from communication style to skill performance and adherence to protocols.

At the beginning of the placement, students approached these differences with curiosity and used them to identify differences between types of settings and types of nurses. They also realized how work style often changes with experience. While this *identification* occurred spontaneously, some students said they used school assignments as *boundary objects* to *reflect* on the differences in nursing practice they encountered.

When students were granted more responsibility, they had to develop their own ways of working. Initially, they often simply copied the way of working of the supervisor of the day. Although differences could be confusing, the students’ peripheral position in the CoP initially kept them from taking further action on these differences.

*I noted that different wound care was used in one patient. One said that the treatment plan said that the acetic acid had to stay on it for 15 min and then remove it and rinse it with normal NaCl and the other said no, I'll leave it on, because then it can be soak in well during the day. This confused me, but because this was at the beginning of my placement, I didn’tt do much with this. (Jenny).*

Students first acts of deviating from their supervisor’s style of working, concerned strictly following protocols even when the supervisor did not. Students reported being driven by the fear of being blamed or assessed when not following a protocol. When they experienced the safety of discussing patient care with their supervisors, they started experimenting with different ways of working. Eventually, they moved toward personal *transformation* by developing their own way of working based on factors such as efficiency and perception of patient care:

*Well, I also often read on the… [hospital information system] about nursing procedures, for example, and what is said about it in the protocol. And if you see something different, then you can also apply it based on whether I think that's easier than the way I do it now, is it more convenient, is it smarter, does it take less time, is it more hygienic. Those are things you pay attention to. And if so, then you can apply that. If that's easier for you, then stick with it. (Abigail).*

Sometimes, students witnessed nursing practices that violated their ethical standards. They engaged in *reflective* conversations about this, leading to either a strengthening or redefining of their previous perspectives on how patients should be treated:

*“So, I noticed that they actually didn't listen to the patient and that they continued with the procedure anyway. And that went beyond my personal limits, so I indicated that afterwards. And I asked why they started mobilizing the patient against his will. And she explained that to me; that didn't change the situation for me. But I found this something that contradicts what I learned at school” (Anne).*

This reflective process helped them develop their identities as nurses, contributing to their personal *transformation*. Often, students integrated different aspects of a both strict/protocol-based approach to nursing and a more empathic approach into their image of the nurse they wanted to become. This transformative process could be accelerated by emotionally loaded experiences:

*“I [have changed my image of the ideal nurse in the sense that] I think it's important to adopt a person-oriented instead of a task-oriented approach. Because of course, I had an unpleasant experience myself. I felt toward my supervisor: gosh I would never want to be like that. “ (Rosalyn).*

### Structuring the learning process: translating requirements and recognizing individual learning preferences

To help students learn across boundaries and keep track of their development, schools provide students with boundary objects, such as competency frameworks and aids to translate these to individual clinical placement settings, as well as tools to collect feedback, assessment standards, and practice assignments. Paradoxically, these tools themselves created additional boundaries as students encountered different ways of understanding and using these standards across settings. Moreover, they sometimes failed to relate school assignments to what actually happened in clinical practice. More advanced students suffered particularly from discrepancies between their required competency level of the curriculum and their actual mastery of patient care in this novel setting.

At the beginning of the clinical placement, the students *identified* different expectations concerning their learning processes. They put effort into *coordinating* school and clinical placement expectations by translating practice standards, bringing parties together, and making sure they received what they needed in their portfolios:

*“I had this idea very early on that you have those traumas, those things. I knew I could not deal with that yet. And then I immediately asked what is expected here from me, and they could not tell me. Then, together with school and practice, I looked at how I can achieve my learning goals here”. (Alexis).*

The focus of these coordinating efforts was on meeting assessment standards: collecting adequate feedback, demonstrating skill mastery, and getting time to work on school assignments. These attempts often stopped when students felt they satisfied all requirements or when they felt they had reached the limits of what they could ask for within their peripheral position in the CoP. Other students persisted until they got what they needed to achieve optimal outcomes from the placement. Students initially tried to solve issues with their direct supervisors. If this did not work, they discussed it with their peers. They then approached the clinical educator, often as a group, who served as a *boundary broker*.

So, while students mainly focused on finding pragmatic solutions to learn in spite of practical boundaries, sometimes the boundaries helped them *reflect* on their learning needs and which objects really helped them, eventually resulting in personal *transformation* as a learner:

*“And, of course, I wrote it down for myself. Just when I had time or something, like oh yes, he said this, I'll write that down. Good tip. Yes, but that's for myself. You hand in those daily evaluations at school and show them, and then you don't do anything with them. But if I have a file for myself with only feedback that I can really apply, I can just take it with me at the next placements and build on it here and there”. (Abigail).*

Opportunities to discuss these experiences with peers, mentors, or in peer review meetings helped students not only make their own learning preferences explicit, but also take the perspective of the supervisor and manage their own expectations for the future. Peers thus seem to broker for each other, as they all know the different contexts and can be of mutual help in turning each other’s experiences into learning.

## Discussion

Nursing students experience different understandings and practices of nursing care and the learning process, both between settings (school, different placements), and within a single placement (different supervisors). This study provides evidence that successfully overcoming these boundary experiences can contribute to professional identity development and competence development in HPE students. During clinical placements, students’ responses to boundary experiences progress: while they initially notice and explore different approaches to nursing care (identification), or try to translate school standards to practice and vice versa (coordination), they subsequently discuss and critically compare these differences (reflection) and eventually try to bring about changes (transformation). The results suggest that personal factors (motivation, confidence, moral convictions) and environmental factors (safety, peers, assessment standards) impact whether these progressions take place. These findings add to the broader boundary-crossing literature by showing how learning mechanisms at the boundary (horizontal learning processes) are interwoven with students’ movement from the periphery to the center of the community of practice (vertical learning processes). The current work can be a basis for studying individual differences in boundary-crossing behavior, as well as learning at the boundaries in other healthcare professions, such as medical students, with different placement structures (Liljedahl et al., 2015).

This study confirms that students experience gaps between their theoretical preparation and the requirements of practice, which temporarily hamper their learning (Greenway et al., 2018). Previous work has considered the transfer of learning from theory to practice as a cyclical process: students learn to apply competence in each novel context and take lessons to repeat this process in the next context (Peters et al., 2017). This cycle suggests that the effective application of theory is the main goal. However, the current results highlight how the *process* of trying to enact can bear learning potential in itself: it forces students to engage in meaningful interactions with staff and peers, helps them reformulate the value of theoretical preparation, and strengthens their skills to collect and interpret information. The struggles they have to overcome may help them develop adaptive expertise (Mylopoulos et al., 2018). Interestingly, aids designed to bridge theory and practice, such as portfolios and practice assignments (boundary objects), can sometimes help students but also generate additional barriers. Thus, the use of boundary objects may become a goal in itself that takes energy away from ‘true’ learning, suggesting a deconstructive struggle (Stoffels et al., 2021; Teunissen & Westerman, 2011). A possible explanation is that the local practice culture has not been completely acknowledged in the design of boundary objects (Sheehan & Wilkinson, 2021). Therefore, theory and practice stakeholders should collaborate to design objects to help students connect the two. An example could be mobile devices in which students find information to prepare for a placement (Kilbrink et al., 2021). How these objects can be designed to promote continuity yet allow for constructive struggle is an area of future study.

In line with the work on learning across the landscapes of practice (Hodson, 2020), this study provides evidence that boundary experiences can contribute to professional identity development: students integrate their experience with different role models into their image of the nurse they want to become. Interestingly, not only differences between settings but also within a setting stimulate reflections about different ways to provide nursing care. This study reveals how the current community of practice plays a role in professional development across boundaries. When the learning climate is safe, students can use their previous experience to critically question staff and experiment with behavior (Levett‐Jones and Lathlean, 2009). At the same time, students’ subordinate positions and the fact that they will be assessed will always be a driving force toward complying with their supervisors’ practice (Bagnasco et al., 2017). Students are likely to openly address their boundary experiences when they feel that their ethical standards are violated. Previous studies showed that behavior upon malpractice was affected by students’ ability to address them, as well as its perceived effect (Mak‐van der Vossen et al., 2018). Therefore, students’ professional development, as well as the ward, would benefit from occasions in which groups of students are invited to openly discuss their experiences in light of their previous experiences and internalized standards (Jack et al., 2021; Kemp et al., 2021). Additionally, HPE curricula should offer students the opportunity to reflect on the different role models they encounter outside the community of practice (Wilkins, 2020). Acknowledging that HPE has a horizontal component in which students not only learn things *more* and *better,* but also *differently* can stimulate these reflections. Future studies can help to understand individual differences in students’ behavior toward conflicting role models.

## Limitations

The findings of this study are based on a small group of students in a single academic setting with a strong tradition of clinical education. This may have affected the kind of boundary experiences students reported as well as their subjective experiences of these. We relied on the students’ self-reports, allowing for the selective and subjective reporting of experiences. However, the design forced students to critically examine how their clinical placement was different from what they expected or what they found important, with prompts from their pre-placement interviews. Joining the study and keeping a diary may have made students more aware of their learning, thereby affecting the process of boundary crossing (Cao & Henderson, 2020). As a result of the study design, the frequency of diary fragments varied, thereby not allowing for a longitudinal analysis. Entries in the diary might not have depended only on students’ boundary experiences but also on their time and motivation. (Verma, 2021).

## Conclusions

Nursing students experience boundaries between the various contexts in which they learn with respect to theory, skill performance, professional behavior, and expectations of the learning process. When the basic requirements of psychological safety are met within the practice setting, students are able to convert these boundary experiences into meaningful learning situations. Thus, boundary experiences can contribute to a better understanding of healthcare and to a more refined image of the professionals students want to become. Accidentally, students can use their experiences to transform the practice itself, although this can be difficult because of their temporary position within the community of practice. Boundaries cannot and should not be removed in HPE. To help HPE students make sense of their boundary experiences, they should be supported to reflect on different ways of practice outside the ward and to be able to access and integrate different resources.

## Appendix 1 Types of boundaries, boundary experiences, learning mechanisms and peripheral experiences

**Table:**

Type of boundary	Boundary experience	Learning mechanism
Delivery of nursing care (total of experiences mentioned: 121; examples of these experiences were mentioned by all of 14 participants)	Difference between current placement and school contents/ previous placements in understanding nursing care (eg skills, theory about pathologies, procedures)	*Identification*: comparing clinical contexts to identify (inherent) differences
		*Identification*: comparing school contents with practice to identify (inherent) differences
		*Coordination*: collecting information on the ward, asking questions, applying information of the ward in school assignments
		*Coordination*: asking how nursing care should be conducted in the current setting to make sure expectations are met
		*Reflection*: comparing and experimenting with different ways of working and thinking about underlying perspectives
		*Transformation*: trying to facilitate filling knowledge and skills gaps for future students
		*Transformation*: Trying to alter practice based on contemporary theory
	Difference between current placements and school contents/ previous placements in nursing behaviour in terms of approaching patients, approaching each other and prioritizing work	*Identification* Comparing possible different ways of conducting nursing care
		*Coordination*: working according to protocols to make sure they can’t be blamed
		*Reflection*: discussing patient care to strengthen or change their perspective
		*Transformation*: integrating different approaches to nursing care into their professional identity
	Differences between supervisors in delivering patient care	*Identification*: comparing different possible ways of conducting nursing care
		*Coordination*: adjusting own working style to the supervisor of the day
		*Transformation*: integrating different working styles into their professional identity
Understanding of the learning process (total of experiences mentioned: 155; examples of these experiences were mentioned by all of 14 participants)	Difference between current placement and school standards about time that can be devoted to ‘learning’ / skills that can be practiced/ learning opportunities granted	*Coordination*: discussing expectations based on requirements from school; trying to align expectations
	Difference between current placement and school standards/ previous placements in giving feedback, formulating learning goals	*Coordination* translating school requirements and assessment forms to the ward
		*Coordination*: taking practical arrangements to meet al requirements
		*Reflection* comparing different approaches to learning and recognizes own learning preferences
		*Transformation*: addressing issues to the ward to improve learning experiences for future students
	Difference between current placement and school standards/ previous placements or between supervisors in independency/level that is expected from the student	*Coordination*: discussing expectations based on requirements from school,
	Difference between supervisors in the current placement in expectancies regarding the learning process and students’ independency	*Coordination*: aligning supervision by making clear expectancies and achievements; arranging they work with preferred supervisors only

**Peripheral experiences** total of experiences mentioned: 56; examples of these experiences were mentioned by all of 14 participants.

**Table:**

Rumors/warning that the placement will be strict/demanding (before placement starts)
Supervisors making clear they don’t like supervising students
Information not being shared with students
Students not being included in celebrations and presents
Students not being asked about their wishes and desires
Individual supervisors not making time for students

## References

[CR1] Akkerman SF (2011). Learning at boundaries. International Journal of Educational Research.

[CR2] Akkerman SF, Bakker A (2011). Boundary crossing and boundary objects. Review of Educational Research.

[CR3] Akkerman SF, Bakker A (2012). Crossing boundaries between school and work during apprenticeships. Vocations and Learning.

[CR4] Akkerman S, Bruining T (2016). Multilevel boundary crossing in a professional development school partnership. Journal of the Learning Sciences.

[CR5] Alzayyat A, Al-Gamal E (2014). A review of the literature regarding stress among nursing students during their clinical education. International Nursing Review.

[CR6] Bagnasco A, Timmins F, de Vries JMA, Aleo G, Zanini M (2017). Understanding and addressing missed care in clinical placements — Implications for nursing students and nurse educators. Nurse Education Today.

[CR7] Barry M, Kuijer-Siebelink W, Niewenhuis LA, Scherpbier N (2020). Professional development arising from multiple-site workplace learning: Boundary crossing between the education and clinical contexts. BMC Medical Education.

[CR8] Bergman E, de Feijter J, Frambach J, Godefrooij M, Slootweg I (2012). AM last page: A guide to research paradigms relevant to medical education. Academic Medicine.

[CR9] Bernabeo EC, Holtman MC, Ginsburg S, Rosenbaum JR, Holmboe ES (2011). Lost in transition: The experience and impact of frequent changes in the inpatient learning environment. Academic Medicine.

[CR10] Berndtsson I, Dahlborg E, Pennbrant S (2020). Work-integrated learning as a pedagogical tool to integrate theory and practice in nursing education – An integrative literature review. Nurse Education in Practice.

[CR11] Bronkhorst LH, Akkerman SF (2016). At the boundary of school: Continuity and discontinuity in learning across contexts. Educational Research Review.

[CR12] Brown ME, Anderson K, Finn GM (2019). A narrative literature review considering the development and implementation of longitudinal integrated clerkships, including a practical guide for application. Journal of Medical Education and Curricular Development.

[CR13] Cao, X. & Henderson, E. F. (2020). The interweaving of diaries and lives: diary-keeping behaviour in a diary-interview study of international students’ employability management. Qualitative Research, 1468794120920260.

[CR14] Cruess RL, Cruess SR, Steinert Y (2018). Medicine as a community of practice: Implications for medical education. Academic Medicine.

[CR15] Dornan T, Tan N, Boshuizen H, Gick R, Isba R (2014). How and what do medical students learn in clerkships? Experience based learning (ExBL). Advances in Health Sciences Education.

[CR16] Engeström, Y., Engeström, R. & Kärkkäinen, M. (1995). Polycontextuality and boundary crossing in expert cognition: Learning and problem solving in complex work activities. Learning and instruction.

[CR17] Filep CV, Turner S, Eidse N, Thompson-Fawcett M, Fitzsimons S (2018). Advancing rigour in solicited diary research. Qualitative Research.

[CR18] Gassas R (2021). Sources of the knowledge-practice gap in nursing: Lessons from an integrative review. Nurse Education Today.

[CR19] Giacomini MK, Cook DJ (2000). Users' guides to the medical literature: Qualitative research in health care a Are the results of the study valid?. Evidence-Based Medicine Working Group Jama.

[CR20] Greenway K, Butt G, Walthall H (2018). What is a theory-practice gap? An exploration of the concept. Nurse Education in Practice.

[CR21] Hazen AC, de Groot E, de Bont AA, de Vocht S, de Gier JJ (2018). Learning through boundary crossing: professional identity formation of pharmacists transitioning to general practice in the Netherlands. Academic Medicine.

[CR22] Henderson A, Cooke M, Creedy DK, Walker R (2012). Nursing students' perceptions of learning in practice environments: A review. Nurse Education Today.

[CR23] Hirsh DA, Ogur B, Thibault GE, Cox M (2007). " Continuity" as an organizing principle for clinical education reform. New England Journal of Medicine.

[CR24] Hodson N (2020). Landscapes of practice in medical education. Medical Education.

[CR25] Jack K, Levett-Jones T, Ylonen A, Ion R, Pich J (2021). “Feel the fear and do it anyway” nursing students’ experiences of confronting poor practice. Nurse Education in Practice.

[CR26] Jacobs G (2017). ‘A guided walk in the woods’: Boundary crossing in a collaborative action research project. Educational Action Research.

[CR27] Kemp C, van Herwerden L, Molloy E, Kleve S, Brimblecombe J (2021). How do students offer value to organisations through work integrated learning?.

[CR28] Kilbrink, N., Enochsson, A.-B., Andersén, A. & Ådefors, A. (2021). Teachers’ use of digital boundary objects to connect school and workplace-based learning in dual vocational education. (In, Developing Connectivity between Education and Work. (pp. 119–136). Routledge)

[CR29] Levett-Jones T, Lathlean J (2009). The ascent to competence conceptual framework: An outcome of a study of belongingness. Journal of Clinical Nursing.

[CR30] Liljedahl M, Boman LE, Falt CP, Bolander Laksov K (2015). What students really learn: Contrasting medical and nursing students' experiences of the clinical learning environment. Advances in Health Sciences Education: Theory and Practice.

[CR31] Mak-van der Vossen M, Teherani A, Van Mook WN, Croiset G, Kusurkar RA (2018). Investigating US medical students' motivation to respond to lapses in professionalism. Medical Education.

[CR32] Mesker P, Wassink H, Akkerman S, Bakker C (2018). Differences that matter: Boundary experiences in student teachers’ intercultural learning. International Journal of Intercultural Relations.

[CR33] Mylopoulos M, Steenhof N, Kaushal A, Woods NN (2018). Twelve tips for designing curricula that support the development of adaptive expertise. Medical Teacher.

[CR34] O'Brien B, Cooke M, Irby DM (2007). Perceptions and attributions of third-year student struggles in clerkships: Do students and clerkship directors agree?. Academic Medicine.

[CR35] O'Brien BC, Harris IB, Beckman TJ, Reed DA, Cook DA (2014). Standards for reporting qualitative research: A synthesis of recommendations. Academic Medicine.

[CR36] Olmos-Vega FM, Dolmans DHJM, Guzmán-Quintero C, Echeverri-Rodriguez C, Teunnissen PW (2019). Disentangling residents’ engagement with communities of clinical practice in the workplace. Advances in Health Sciences Education.

[CR37] Peters S, Clarebout G, Diemers A, Delvaux N, Verburgh A (2017). Enhancing the connection between the classroom and the clinical workplace: A systematic review. Perspectives on Medical Education.

[CR38] Roxburgh M, Conlon M, Banks D (2012). Evaluating hub and spoke models of practice learning in Scotland, UK: A multiple case study approach. Nurse Education Today.

[CR39] Salisu WJ, Dehghan Nayeri N, Yakubu I, Ebrahimpour F (2019). Challenges and facilitators of professional socialization: A systematic review. Nursing Open.

[CR40] Sheehan D, Wilkinson TJ (2021). Widening how we see the impact of culture on learning, practice and identity development in clinical environments. Medical Education.

[CR41] Stoffels M, Peerdeman SM, Daelmans HE, Ket JC, Kusurkar RA (2019). How do undergraduate nursing students learn in the hospital setting? A scoping review of conceptualisations operationalisations and learning activities. British Medical Journal Open.

[CR42] Stoffels M, van der Burgt SM, Stenfors T, Daelmans HE, Peerdeman SM (2021). Conceptions of clinical learning among stakeholders involved in undergraduate nursing education: A phenomenographic study. BMC Medical Education.

[CR43] Teunissen PW, Westerman M (2011). Opportunity or threat: The ambiguity of the consequences of transitions in medical education. Medical Education.

[CR44] Thrysoe L, Hounsgaard L, Dohn NB, Wagner L (2010). Participating in a community of practice as a prerequisite for becoming a nurse - trajectories as final year nursing students. Nurse Education in Practice.

[CR45] van Dijk I, Lucassen PLBJ, van Weel C, Speckens AEM (2017). A cross-sectional examination of psychological distress, positive mental health and their predictors in medical students in their clinical clerkships. BMC Medical Education.

[CR46] Verma, A. (2021). Using audio-diaries for research and education: AMEE Guide No. 144. Medical Teacher, 1–7. 10.1080/0142159X.2021.1972954 34499842

[CR47] Weeks K, Coben D, Lum G, Pontin D (2017). Developing nursing competence: Future proofing nurses for the changing practice requirements of 21st century healthcare. Nurse Education in Practice.

[CR48] Wenger-Trayner, E. & Wenger-Trayner, B. (2014). Learning in a landscape of practice: A framework. (In, Learning in landscapes of practice. (pp. 13–29). Routledge)

[CR49] Wilkins EB (2020). Facilitating professional identity development in healthcare education. New Directions for Teaching and Learning.

[CR50] Williamson GR, Plowright H, Kane A, Bunce J, Clarke D (2020). Collaborative learning in practice: A systematic review and narrative synthesis of the research evidence in nurse education. Nurse Education in Practice.

[CR51] Worley P, Couper I, Strasser R, Graves L, Cummings B-A (2016). A typology of longitudinal integrated clerkships. Medical Education.

